# The Relationship Between Anemia and Parathyroid Hormone Levels in Patients With Kidney Failure Undergoing Hemodialysis Treatment in Georgia

**DOI:** 10.7759/cureus.64021

**Published:** 2024-07-07

**Authors:** Elene Shavgulidze, Nikoloz Papiashvili, Goga Jatchvadze, Elisabed Chikobava, Krithika Mennambath, Adil Baig, Nithesh Hariharan, Irma Tchokhonelidze

**Affiliations:** 1 American MD Program, Tbilisi State Medical University, Tbilisi, GEO; 2 Faculty of Medicine, Tbilisi State Medical University, Tbilisi, GEO; 3 Nephrology, Ingorokva High Medical Technology University Clinic, Tbilisi, GEO

**Keywords:** hyperparathyroidism, kidney failure, anemia, parathyroid hormone, hemodialysis

## Abstract

Introduction: Chronic kidney disease (CKD) and its associated complications, such as anemia and secondary hyperparathyroidism (SHPT), pose significant challenges to global healthcare systems. This study explores the demographic and clinical characteristics of 284 kidney failure (KF) patients undergoing hemodialysis, in an effort to shed light on the possible association between anemia and SHPT. A proven connection between the two could theoretically influence the management plans for CKD patients, with the hopes of achieving lower morbidity and/or mortality in this patient group.

Methods: A retrospective, cross-sectional, real-world data analytical study was conducted at a hemodialysis center in Tbilisi, Georgia, encompassing a sample size of n = 284 patients on maintenance hemodialysis. The data analyzed was extracted from patients’ medical records.

Results: According to our results, the prevalence of anemia was strikingly high at 82.04%, underlining its substantial burden within this patient population. Our analysis revealed a notable systemic association between anemia and SHPT, particularly when considering hemodialysis vintage. However, our final analysis model revealed no statistically significant association between anemia and intact parathyroid hormone (iPTH) levels.

Conclusion: Our study revealed a significant systemic relationship between anemia and SHPT when hemodialysis duration was considered, despite initial analyses showing no direct association. Future research should focus on longitudinal and multi-center studies to better understand this relationship, aiming to enhance the care and management of CKD patients on hemodialysis.

## Introduction

The coexistence of anemia and secondary hyperparathyroidism (SHPT) in patients with kidney failure (KF) undergoing hemodialysis (HD) has been a subject of significant research interest. Numerous studies have explored the potential relationship between these two conditions, yet the results have often been inconclusive. Moreover, it is noteworthy that SHPT, despite being a critical aspect of chronic kidney disease (CKD), remains relatively underrecognized as a potential contributor to the development of anemia in these patients. To explore this association, we must first consider the multifaceted nature of both anemia and SHPT in the context of CKD.

Renal anemia, a common complication in patients with CKD, has a multitude of underlying causes. These encompass factors such as decreased production and resistance to erythropoietin, a shortened lifespan of red blood cells (RBCs), and bone marrow fibrosis. SHPT is hypothesized to be a potentially important cause of renal anemia in CKD patients. It is mainly characterized by high serum intact parathyroid hormone (iPTH) levels, parathyroid gland hyperplasia, and changes in mineral metabolism, mainly hypocalcemia and hyperphosphatemia [1]. The interplay between iPTH, calcium, and phosphorus plays a pivotal role in mineral bone metabolism in CKD patients, influencing various physiological processes. Calcium is essential for muscle contraction, nerve impulse transmission, and blood clotting, while phosphorus is integral to energy metabolism and bone health. iPTH and vitamin D act as regulators, maintaining the balance of calcium and phosphorus in the bloodstream.

A review of existing literature reveals two major proposed mechanisms by which SHPT may decrease serum hemoglobin (Hgb) levels in KF patients. According to the first mechanism, an increased iPTH level leads to a significant decrease in erythropoietin production, consequently reducing erythropoiesis and decreasing serum Hgb levels in KF patients [2]. This effect has been substantiated by studies demonstrating that parathyroidectomy can reduce the required maintenance dose for erythropoiesis-stimulating agents (ESAs) in CKD patients [3]. The second mechanism hinges on the hemolytic effects of elevated iPTH due to the effects of calcium, which increases the osmotic pressure and fragility of RBCs [4]. Kalantar-Zadeh et al. endorsed this by showing that higher iPTH levels were independently associated with ESA hyporesponsiveness [5]. Similarly, another study reported a modest but significant association between higher iPTH levels and decreased erythropoietic response [6]. It is likely that these mechanisms collectively contribute to the development of renal anemia in CKD patients.

Few studies have directly compared anemia and iPTH levels in hemodialysis (HD) patients, and the results remain insufficient to validate the association between SHPT and anemia. As an example, Bukhari et al. found no statistically significant association between hyperparathyroidism and anemia [7], while Chutia et al. revealed a statistically significant negative correlation between iPTH and Hgb levels [8]. Although considerable research has gone into understanding and improving the medical outcomes associated with these complications of CKD, limited advances have been made. A definitive connection between anemia and SHPT could serve as a foundation for more effective treatment plans [9].

The main goal of this research was to define the relationship between anemia and SHPT in patients undergoing hemodialysis treatment. By understanding the specific relationship between anemia and PTH levels in HD patients, we have the potential to develop more targeted strategies for managing anemia, with the potential to prevent complications and improve treatment outcomes, especially in low-resource countries.

## Materials and methods

Study design and clinical settings

We adopted a retrospective, cross-sectional design, covering the period from September 2023 to December 2023. Data pertinent to the investigation was exclusively sourced from patient medical records maintained at the hemodialysis center of High Technology Medical Center University Clinic, Tbilisi, Georgia.

Selection criteria

The minimum sample size (n=197) was calculated by using Raosoft, an online sample size calculator, keeping power at 50%, level of significance at 5%, and confidence interval at 95%. In our study, we focused on individuals aged 18 years and older who had undergone maintenance hemodialysis for a minimum of three months. Rigorous exclusion criteria were applied, excluding participants who had undergone renal transplantation, died within the initial three months of initiation, or ceased hemodialysis treatment post the three-month threshold. These criteria were aimed to ensure the homogeneity and relevance of the selected cohort for the investigation. Simple random sampling was employed to recruit study subjects. 

Data collection and screening

We collected our information by reviewing the medical records of hemodialysis patients at the dialysis center located in the aforementioned hospital. We sifted through the records, checking against a predefined set of inclusion and exclusion criteria. Upon successful screening, we organized the data into a database in an Excel sheet.

The study included patients on maintenance hemodialysis from both urban and rural areas of Georgia. The data gathered includes demographics, date of first hemodialysis, number of years on hemodialysis, and laboratory profiles of Hgb, iPTH, calcium, phosphorus, and vitamin D (25(OH)D3). It should be noted that some data could not be collected for certain patients and the results described below are reflective of this.

In our examination of KF patients, we established cutoff values for serum iPTH levels according to the Kidney Disease: Improving Global Outcomes (KDIGO) 2017 guidelines [10]. Specifically, levels below 130 pg/mL were categorized as below the target serum iPTH level, levels ranging from 130 to 600 pg/mL were classified as the target serum iPTH level, and levels exceeding 600 pg/mL were identified as above the target serum iPTH level or indicative of hyperparathyroidism. Similarly, a cutoff value for hemoglobin in terms of anemia was referred from the World Health Organization (WHO) [11]. Anemia was defined as a hemoglobin level below 12 g/dL in women and below 13 g/dL in men. These criteria were employed to categorize individuals within our study cohort for further analysis.

Statistical analysis

We analyzed the data using GraphPad Prism 9.5.0 (GraphPad Software, Boston, MA). For continuous variables, we reported the key parameters: the mean, standard deviation, median, minimum, and maximum values. Meanwhile, categorical variables are presented as frequencies and percentages. We utilized unpaired t-tests to compare Hgb and iPTH levels across different age groups and sexes. We conducted correlation analysis to thoroughly explore connections between various factors, such as Hgb, iPTH, Ca, Phosphorus, vitamin D, and hemodialysis duration. To evaluate the possible associations between various factors and anemia, Fisher’s exact Chi-square test was used for categorical variables. Additionally, a chi-square test for trend was performed to investigate the relationship between Hgb, iPTH, and hemodialysis vintage (<1 year, 1-5 years, 5-10 years, and >10 years).

To assess the predictors impacting Hgb levels, a multiple linear regression model was used, taking into account factors such as iPTH, calcium, phosphorus, vitamin D, age, and HD duration. To investigate potential differences in Hgb means across distinct groups categorized by levels of iPTH, an analysis of variance (ANOVA) was conducted.

The above-mentioned analyses were done to find the correlation between various iPTH levels and Hgb. The patients were classified into three iPTH groups: iPTH < 130, 130 <= iPTH <600, and iPTH>= 600. All patients had iron studies within the protocol-defined range (KDIGO Anemia Guideline 2012) [12]. The mean dosage of Epo-alfa was 50 IU/kg. We excluded the patients with ongoing infection. These comprehensive analyses allowed us to explore the intricate relationships and factors impacting hemoglobin levels in our study population.

## Results

**Figure 1:**
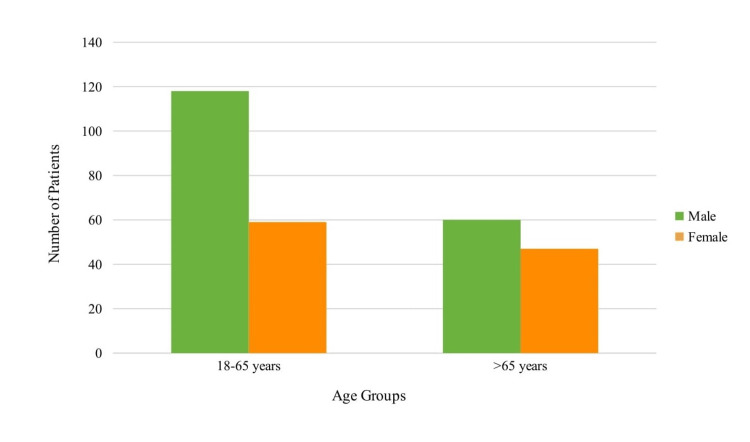
Graphical representation of age and gender distribution in the cohort

**Figure 2:**
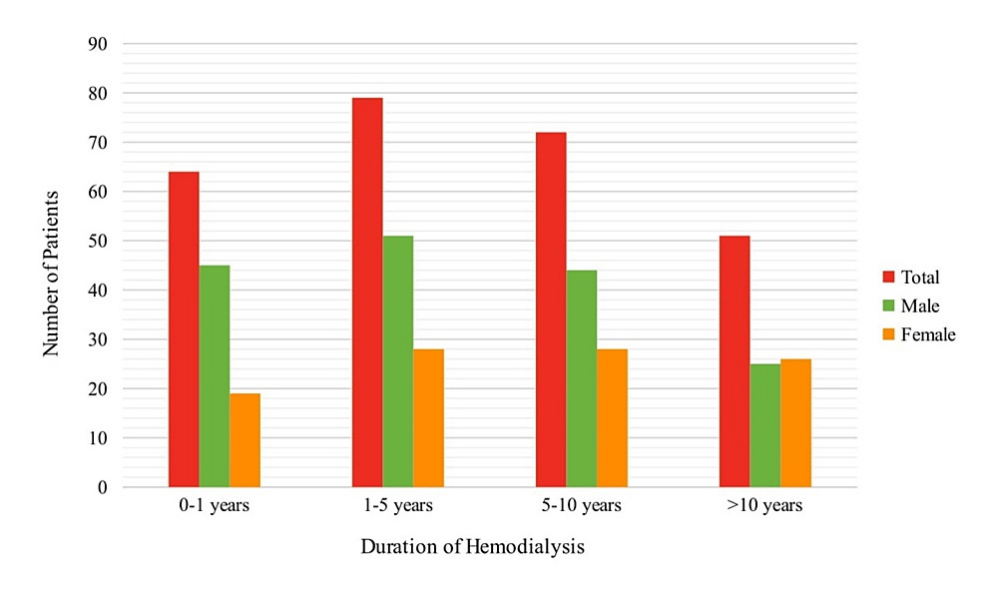
Graphical representation of patient distribution by duration of hemodialysis

A total of 284 patients were recruited into our study with a mean age of 61±13 years (Figure 1). Patients were also categorized based on HD duration, with ranges less than 1 year, 1-5 years, 5-10 years, and more than 10 years (Figure 2).

Among our study sample, a high prevalence of anemia was observed, seen in 82.04% of patients. Analysis of iPTH levels (n=245) demonstrated iPTH levels above the target range (>600 pg/mL) in 61 (24.89%) patients. For a comprehensive overview of these findings, please refer to Table 1.

**Table 1:** Characteristics of the study sample iPTH: Intact parathyroid hormone

Patient Characteristics	n (%)
Male	178 (62.68%)
Female	106 (37.32%)
Mean Age	61 ± 13 years
18-65 years	177 (62.32%)
>65 years	107 (37.68%)
Duration of Hemodialysis 0-1 years	64 (24.06%)
Duration of Hemodialysis 1-5 years	79 (29.70%)
Duration of Hemodialysis 5-10 years	72 (27.07%)
Duration of Hemodialysis >10 years	51 (19.17%)
Total patients with anemia	233 (82.04%)
Male patients with anemia	156 (87.64%)
Female patients with anemia	77 (72.64%)
Total patients with iPTH below target range (<130 pg/mL)	45 (18.37%)
Total patients with iPTH target range (130-600 pg/mL)	140 (57.14%)
Total patients with iPTH above target range (>600 pg/mL)	60 (24.49%)

The results of correlation analysis between hemoglobin levels and various covariates are presented in Table 2. We identified a weak but statistically significant correlation between serum hemoglobin levels and duration of hemodialysis (r = 0.266, 95% CI: 0.141 - 0.382, p < 0.001). No correlation was found between hemoglobin levels and patient age (p = 0.506). Furthermore, we observed a very weak correlation between serum calcium levels and Hgb levels (r = 0.136, 95% CI: 0.011 - 0.257, p = 0.033). We also explored the relationship between serum vitamin D (25(OH)D3) levels and hemoglobin levels, revealing a weak correlation (r = 0.255, 95% CI: 0.112 - 0.388, p < 0.001). However, the correlation between iPTH and serum hemoglobin was found to be statistically insignificant (p = 0.552).

**Table 2:** Correlation between hemoglobin and covariates P (Phosphorus); iPTH (Intact parathyroid hormone)

Covariates	Pearson’s correlation coefficient (r)	95% CI	p Value
Age	- 0.042	- 0.167 – 0.083	0.506
Duration of Hemodialysis	0.266	0.141 – 0.382	<0.001
Ca	0.136	0.011 – 0.257	0.033
P	- 0.050	- 0.175 – 0.076	0.433
iPTH	- 0.038	- 0.163 – 0.088	0.552
Vitamin D (25(OH)D_3_)	0.255	0.112 – 0.388	<0.001

Additionally, when examining the correlation between iPTH levels and the duration of HD, we found a negligible positive correlation (r = 0.05) with a p-value of 0.4. Additionally, comparisons based on age groups and sexes did not yield statistically significant differences in iPTH levels. Our comparison of hemoglobin and iPTH levels between different age groups through unpaired t-tests did not reveal any statistically significant differences. Similarly, when these comparisons were conducted between different sexes, no statistically significant differences were observed.

The Chi-square test for trend was performed to investigate the potential association between hemoglobin, iPTH, and HD duration across ordered groups. The analysis revealed a statistically significant trend among these variables (χ² = 12.41, p = 0.0061). Additionally, we conducted a Chi-square test to explore the potential association between anemia and iPTH levels across ordered groups. The analysis yielded a p-value of 0.052, which is insufficient to establish a significant association between anemia and iPTH levels.

A multiple linear regression analysis was conducted to examine the relationship between hemoglobin levels and various predictors, including PTH, age, calcium, phosphorus, sex, and vitamin D. The results are summarized in Table 3. The overall model was statistically significant (p = 0.0014). Among the predictors, calcium and vitamin D were found to be significant. Specifically, for each unit increase in calcium, hemoglobin levels increased by 1.960 units on average (p = 0.0038). Similarly, for each unit increase in vitamin D, hemoglobin levels increased by 0.01729 units on average (p = 0.0014). Other predictors, including PTH, age, phosphorus, and sex, did not show significant associations with hemoglobin levels. The intercept was also statistically significant, indicating that the mean hemoglobin level, when all predictors are zero, is 6.252 (p = 0.0001).

**Table 3:** Multiple linear regression analysis summary ns: Not significant (p > 0.05); * * *: Statistically significant at different levels (e.g., *** p < 0.001, ** p < 0.01); PTH: parathyroid hormone

Variable	Estimate	Standard Error	95% CI	t-value	p-value	Significance
Intercept	6.252	1.603	[3.088 - 9.416]	3.901	0.0001	***
PTH	-0.0004263	0.0002858	[-0.0009905 - 0.0001379]	1.491	0.1377	ns
Age	0.0005416	0.009906	[-0.01901 - 0.02010]	0.05467	0.9565	ns
Calcium (Ca)	1.96	0.6671	[0.6429 - 3.276]	2.938	0.0038	**
Phosphorus	0.1885	0.2355	[-0.2763 - 0.6534]	0.8006	0.4245	ns
Sex	-0.2387	0.2784	[-0.7882 - 0.3108]	0.8575	0.3923	ns
Vitamin D (Vit D)	0.01729	0.005334	[0.006761 - 0.02782]	3.242	0.0014	**

**Figure 3:**
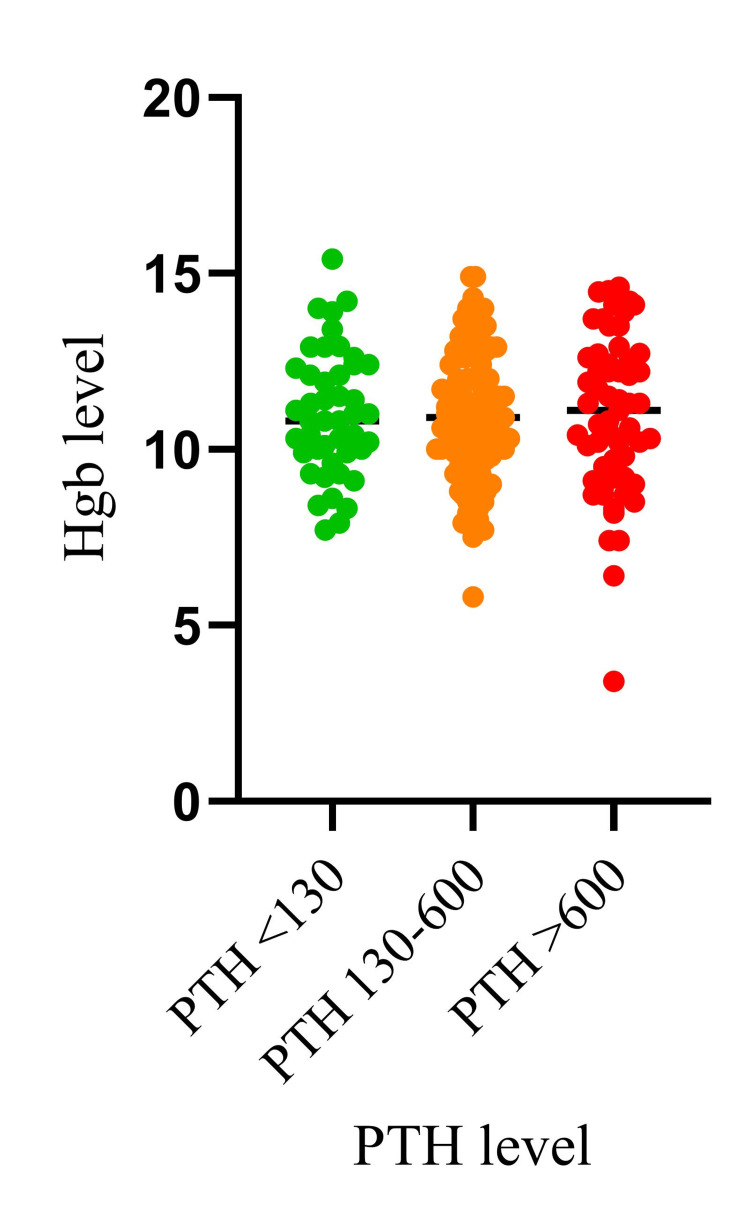
Comparison of mean hemoglobin levels across different PTH level groups PTH: Parathyroid hormone

Finally, a one-way ANOVA test was conducted to compare the mean hemoglobin levels across different parathyroid hormone level groups. Results indicated no statistically significant difference in the mean hemoglobin levels across the different PTH level groups (p = 0.9917). For visualization of these findings, please refer to Figure 3.

## Discussion

In light of our findings, several crucial insights emerge regarding the demographic and clinical characteristics of patients undergoing hemodialysis for CKD. Notably, we observed a strikingly high prevalence of anemia within our cohort, affecting 82.04% of patients. This finding underscores the substantial burden of this hematologic complication within the hemodialysis population. This aligns with existing literature emphasizing the increased susceptibility to anemia in CKD patients due to factors such as erythropoietin deficiency, iron metabolism disturbances, and chronic inflammation induced by renal dysfunction leading to alterations in ferritin, hepcidin, and transferrin [13]. Our study has gauged anemia in KF patients on HD maintenance and determined the correlation of anemia with covariates such as demographics, SHPT, and HD duration in 284 maintenance hemodialysis patients in Georgia, hemodialysis center in High Technology Medical Center University Clinic.

As per our results, the mean age of KF patients undergoing hemodialysis treatment in our cohort is 61±13 years. Other large-scale cross-sectional studies have also yielded similar results. Bonenkamp et al. [14] reported a mean age of 62.5±14, while Nitta et al. [15] found the average age to be 68.43 years. Anemia prevalence was higher in males compared to females in our study. This finding is consistent with a cross-sectional study conducted in Mexico, which assessed the factors influencing anemia in hemodialysis patients [16]. However, some cross-sectional studies reported that women with CKD were more likely to develop anemia compared to men [17]. Mean hemoglobin values were not significantly different (p = 0.7168) in the 18-65 years and >65 years age groups in our cohort.

Existing literature demonstrates that within individuals with KF, the excessive production of iPTH causes alterations in mineral and bone metabolism, ultimately contributing to the development of anemia. The association between the prevalence of anemia and SHPT has been proposed by multiple authors [1,7-8,18], alongside hypotheses for potential pathophysiological mechanisms between the two [2]. As per our study, the association between elevated iPTH levels and hemoglobin was not significant. Both correlation analysis and univariate regression indicated a non-significant association between iPTH levels exceeding 600 pg/mL and anemia. Our results were comparable to a study by Bukhari et al. [7] that described no significant association between anemia and iPTH levels. However, Chutia et al. [8], along with Soleymanian et al. [18], reported a statistically significant reverse correlation between iPTH and hemoglobin levels.

A statistically significant association was observed between hemoglobin and iPTH when HD duration is taken into account (χ² = 12.41, p = 0.0061), suggesting the existence of a discernible trend among these variables, signifying a relationship that goes beyond random chance. However, further exploration is required to elucidate the direction and strength of this relationship.

Regarding mineral and bone disorders in CKD, our identification of significant correlations between iPTH levels, serum calcium, and serum phosphorus aligns with established pathophysiological mechanisms documented by Arora et al. [19] and corroborates the intricate interplay between these parameters in CKD-mineral bone disorder (CKD-MBD). However, the lack of a robust association between iPTH levels and HD duration in our study contrasts with the findings of Yu et al. [20], who reported a clear influence of treatment duration on iPTH regulation. Their results showed that the mean duration of dialysis was longer in the low iPTH group than in the SHPT group. This discrepancy might highlight the variability in iPTH responses among diverse patient cohorts or could emphasize the need for more extensive longitudinal studies to understand this relationship comprehensively.

Notably, a statistically significant association between hemoglobin level and HD duration was found in correlation analysis (r = 0.266, p < 0.001). This implies that patients who have been on maintenance hemodialysis for a longer period of time are more likely to have a higher hemoglobin level. However, while our study reflects existing literature concerning anemia prevalence, the association between hemoglobin levels and HD duration warrants further discussion in light of conflicting findings reported in earlier studies. Contrary to the observations of Zhu et al. [21] who noted a decline in hemoglobin levels with prolonged HD duration, our study identified a positive correlation between these variables. These divergent results suggest potential variability in hematologic responses among different patient populations or underline the influence of additional factors, such as variations in treatment protocols or patient demographics, necessitating deeper exploration for clarification.

It should be noted that all patients participating in our study were started on ESA therapy with epoetin alfa upon hemodialysis initiation, with a weekly dosage ranging from 1200 to 15000 units. This treatment protocol was constructed according to the Kidney Disease: Improving Global Outcomes (KDIGO) 2012 guidelines, which recommend starting an ESA on an individual basis [12]. ESAs are administered to most hemodialysis patients with hemoglobin (Hb) <10 g/dL who do not have iron deficiency [22]. Several studies comparing pre- and post-dialysis hemoglobin levels revealed significantly higher hemoglobin levels post-dialysis in the acute setting [23], further supporting our results, though in the chronic setting. Since decreasing serum iPTH levels have been shown to lead to an increase in serum immunoreactive erythropoietin levels [24], ESA therapy could potentially be masking the effects of this relationship, prompting further investigation.

Our study contained multiple limitations that should be acknowledged. The sample size used was relatively small, which may limit the generalizability of results to the general HD population. In addition, data was collected from only a single hemodialysis center, which may not accurately represent this patient demographic. Furthermore, because of its cross-sectional and observational nature, the present study may not provide definite information about the causal relationship. While causality cannot be definitively established, the observed relationships and trends are valuable for generating hypotheses and guiding further research. Moreover, the retrospective nature of the study posed inherent limitations, as it relied on previously collected data, leading to potential biases and limitations in data completeness. Furthermore, the data collected for our study reflects the latest measurement of each variable, rather than considering the mean of measurements over a specified period, such as the last six months or one year. This may have overlooked temporal variations in parameters, possibly impacting the accuracy of the results. 

We recommend that future studies take into account the effect of various medications on hemoglobin levels, notably cinacalcet, which is frequently used in CKD patients undergoing hemodialysis therapy to regulate parathyroid hormone and calcium homeostasis. This was originally planned to be included in our study but was ultimately discarded due to a lack of patients undergoing therapy with cinacalcet, attributed to lack of availability. Further studies should also focus on the variability in hemoglobin levels among patients who have undergone parathyroidectomy, compared to those who have not.

These limitations warrant caution in interpreting the findings and emphasize the need for further investigations encompassing larger, more diverse cohorts and employing prospective, multi-center designs to overcome these constraints and enhance the robustness and reliability of future studies in this field.

## Conclusions

Our study explored the association between anemia and secondary hyperparathyroidism (SHPT) in hemodialysis patients. While initial analyses did not show a direct link between elevated iPTH levels and anemia, a significant relationship emerged when hemodialysis duration was considered. These findings offer a clearer understanding of the complex relationship between anemia and SHPT, highlighting the challenges in managing these conditions in CKD patients. The significant relationship we discovered, when considering hemodialysis duration, suggests that future research should focus on longitudinal studies and collaborative efforts among various medical centers. Such research will be essential in further elucidating the interactions between anemia and SHPT, ultimately leading to improved care and management strategies for CKD patients undergoing maintenance hemodialysis.
